# Clinical Trends and Novel Research Insights into Testicular and Penile Disorders and Infertility

**DOI:** 10.3390/life14070878

**Published:** 2024-07-15

**Authors:** Uros Bumbasirevic

**Affiliations:** 1 Clinic of Urology, University Clinical Center of Serbia, 11000 Belgrade, Serbia; urosbu@gmail.com; 2 Faculty of Medicine, University of Belgrade, 11000 Belgrade, Serbia

## 1. Introduction

Due to their great heterogeneity with regard to etiopathogenesis, the clinical presentations, diagnostics, treatment approaches, and potential complications of testicular and penile disorders, despite posing a significant public health burden, constitute an immensely complex, highly fascinating, and continually evolving topic in urology. In our Special Issue, titled “Clinical Trends and Novel Research Insights into Testicular and Penile Disorders and Infertility”, we have gathered fundamental, clinical, and translational studies, along with comprehensive reviews, that aim to enhance our existing understanding and drive significant innovations and improvements in these fields. The studies featured in this Special Issue provide distinct contributions to selected topics, ranging from complex surgical reconstructions of congenital genital anomalies [1] and evaluating new possibilities in gender-affirming surgery [2] to exploring potential causes of decline in global fertility and fecundity [3], as well as investigating associations between asthma and male infertility [4] and evaluating the quality of life of men undergoing infertility treatment [5]. Furthermore, additional studies and reviews explore the complexity of genetic bases and the effect of environmental insults on gonadal dysgenesis [6] and testicular cancer [7,8] development, along with investigations into systemic inflammation indices and their potential prognostic significance in testicular cancer [9].

## 2. Highlights from This Special Issue

Bencic et al. retrospectively evaluated long-term aesthetic and functional outcomes of the surgical repair of isolated male epispadias [1]. A satisfactory aesthetic outcome was achieved in majority of the patients evaluated (71.4%). This complex and sophisticated surgical repair process typically included more than two surgical procedures, and unresolved urinary incontinence was detected in one-fifth of the patients, with substantial negative impacts on their quality of life and the need for additional treatment noted. Moreover, the authors highlighted the need for long-term follow-up after puberty due to the possibility of late complications.

In a highly unique original study titled “Penile Microdissection: A Live Donor Feasibility in Feminizing Gender-Affirming Surgery”, Pusica and colleagues presented detailed descriptions and results of penile microvascular dissection, based on penile disassembly principles, in patients receiving gender-affirming vaginoplasties, and they confirmed the feasibility of preserved penile tissue for potential live-donor penile transplantation [2]. The described microdissection technique was associated with preserved corpora cavernosa and anterior urethra, along with good glans volume, indicating a significant step forward in the development of live-donor penile transplantation techniques.

Aitken’s in-depth analysis of the global decline in fertility provides a comprehensive assessment of the mechanisms underlying this rapidly and ubiquitously occurring phenomenon [3]. Aitken implies that fast demographic transitions, influenced by socioeconomic and cultural factors, result in a rapid decline in fertility, while synchronous genetic and epigenetic factors could contribute to a substantial long-term decrease in human fertility and fecundity. Recognizing these mechanisms could guide future policies and strategies aimed at reversing the current decline in fertility rates.

In order to provide additional insight into the decline in male fertility, Pedersen et al. carried out a cross-sectional study to elucidate the relationship between lower sperm count and asthma [4]. This large-scale study revealed a correlation between self-reported asthma and decreased total sperm count and sperm concentration. These findings suggest a potential link between systemic inflammation associated with asthma and impaired testicular function, opening up an intriguing field for further research.

Despite the increasing prevalence of male infertility, the impact of infertility and its treatment on quality of life (QoL) and emotional distress is still underestimated and neglected. Thus, using a thorough, multi-modal examination of males receiving infertility therapy, Cegar et al. found a considerable prevalence of stress, anxiety, and depression [5]. These findings have tremendous potential for developing focused therapies and providing better patient care, with the ultimate goal of improving treatment outcomes and well-being.

Approximately 50% of all 46, XY gonadal dysgenesis cases have an unknown genetic etiology. In light of recent studies, de Oliveira and colleagues evaluated the role of DHX37 and NR5A1 variants in the etiopathogenesis of this significant testicular differentiation disorder [6]. By implementing whole-exome sequencing (WES), the authors provided significant evidence supporting the association of DHX37 and NR5A1 variants with 46, XY gonadal dysgenesis development, thus immensely contributing to the understanding of the underlying genetic causes of this disorder.

The role of polymorphisms of the omega class of glutathione transferases (GSTO1 and GSTO2)—major components of the cellular antioxidative defense system—in relation to the risk of testicular cancer development is unknown. Petrovic and colleagues conducted the first study to analyze the individual, combined, and cumulative effect of specific GSTO polymorphisms (GSTO1rs4925, GSTO2rs156697, and GSTO2rs2297235) and observed a significant link to the elevated risk of developing testicular cancer [7]. This pioneering study paves the way for further investigations in this developing and emerging field.

In order to further elucidate the complex intricacies of testicular cancer etiopathogenesis, Beck and colleagues conducted a comprehensive systematic review and meta-analysis on the role of maternal exposure to smoke during pregnancy and the risk of testicular cancer development in offspring [8]. Although this high-quality review suggested a tendency toward a greater risk of testicular cancer in mothers who were exposed to cigarette smoking throughout their pregnancy, the meta-analysis conducted did not confirm these results. This study provides insightful recommendations for overcoming methodological limitations, inconsistencies, and gaps in future studies on this subject.

Based on the increasing understanding of the interplay between inflammation and neoplastic transformation and progression, multiple systemic indices of inflammation with considerable prognostic and clinical potential have been developed. Janicic et al. conducted an in-depth narrative review that summarized the significance of various systemic inflammation indices in predicting outcomes in patients with testicular and penile cancers [9]. The authors recognized that these complex indices have the potential to complement and enhance the accuracy of existing biomarkers and clinicopathological parameters, leading to better risk assessment and decision making.

## 3. Final Reflections

In conclusion, this Special Issue presents a remarkable collection of multidisciplinary studies, ranging from fundamental to clinical research, encompassing in-depth reviews and meta-analysis, and assembled with the goal of enhancing our understanding of development, clinical assessment, and treatment of male genitalia disorders. While broadening and challenging current paradigms and knowledge, this Special Issue opens the door to novel perspectives and paves the way for important advancements and discoveries in this field.
